# Application of the PJ and NPS evaporation duct models over the South China Sea (SCS) in winter

**DOI:** 10.1371/journal.pone.0172284

**Published:** 2017-03-08

**Authors:** Shaobo Yang, Xingfei Li, Chao Wu, Xin He, Ying Zhong

**Affiliations:** State Key Laboratory of Precision Measuring Technology and Instruments, Tianjin University, Tianjin, China; Tallinn University of Technology, ESTONIA

## Abstract

The detection of duct height has a significant effect on marine radar or wireless apparatus applications. The paper presents two models to verify the adaptation of evaporation duct models in the SCS in winter. A meteorological gradient instrument used to measure evaporation ducts was fabricated using hydrological and meteorological sensors at different heights. An experiment on the adaptive characteristics of evaporation duct models was carried out over the SCS. The heights of the evaporation ducts were measured by means of log-linear fit, Paulus-Jeske (PJ) and Naval Postgraduate School (NPS) models. The results showed that NPS model offered significant advantages in stability compared with the PJ model. According the collected data computed by the NPS model, the mean deviation (MD) was -1.7 m, and the Standard Deviation (STD) of the MD was 0.8 m compared with the true value. The NPS model may be more suitable for estimating the evaporation duct height in the SCS in winter due to its simpler system characteristics compared with meteorological gradient instruments.

## Introduction

Atmospheric optical refraction has attracted considerable attention in the last few years in atmospheric science, physics, and electronics and so on. Nonstandard refraction of electromagnetic radiation in the lower troposphere can cause radio or radar signals to propagate so that the curvature of their path is greater than the earth’s surface curvature. These microwaves may be trapped within this duct layer, which can lead to forming tropospheric duct propagation [1–2]. Evaporation duct can significantly increase (sometimes, by several orders of magnitude) the range of radio transmitters and radar stations when operated in duct environment which has been investigated and confirmed in numerous works [3]. Ducts occasionally support the normal propagation of radar signals and can cause signal distortion and attenuation, which be depended on the atmospheric refractivity and on its close relation to atmospheric factors such as moisture, wind, air and sea surface temperatures (SST). The atmospheric refractivity profile is usually derived from local surface and upper air meteorological data. Researches show that the probability of occurrence for evaporation duct reaches 80% in the SCS. The SCS is not only exceedingly important shipping channels but also a part of One Belt and One Road in the word. Therefore, the exact measurement and forecasting of evaporation duct height (EDH) over the SCS is an extremely important factor for improving the performance of radar and communication systems as they work in the atmosphere duct environment [4–6].

In 2009, Yang K D used NPS model to calculate evaporation duct height based on the National Centers for Environmental Prediction data. The evaporation duct is about 14 m in the north section of SCS in winter [7]. Tian B used PJ model to analysis the evaporation duct of subtropical sea zone and pointed that PJ model is adaptive for estimate the evaporation duct height [8]. Jing J L pointed offered a new model called New model and pointed that the probability of occurrence for evaporation duct is 100% in winter [9]. In 2013, Cheng Y H pointed that the probability of occurrence for evaporation duct height exceeding 10 m is more than 85% in winter over the SCS [10–11].

To some degree, evaporation ducts exist over the ocean at all times, and duct height is a key practical factor. The evaporation duct height can vary from a meter in northern latitudes during winter nights to almost forty meters in equatorial latitudes during summer days. There are numerous methods for measuring evaporation ducts, including direct measurement with a microwave refraction instrument [12], which can incur significant costs due to the required precise control of the vertical height of the instrument. Indirect measurements utilize meteorological gradient instruments based on the iron tower platforms, radiosondes, and retrievals technology. Radiosondes offer the advantage of high resolution, but the measurement continuity of the data are difficult to assure. Retrievals technology includes refractivity from clutter (RFC), GPS retrieval technology, and LIDAR retrieval [13–15]. RFC technology only uses radar and does not require any additional hardware for achieving echo power signals containing scattered and reflected signals over the sea. GPS retrieval technology constructs a model between the GPS delay signals and the atmospheric refraction effect. However, these retrieval techniques are limited by simple exposition, lower resolution, and lack of real-time information. Therefore, evaporation duct models based on iron towers are used widely in practice because of their low cost, high accuracy and real-time data compared with the other methods.

The meteorological gradient instrument used to measure the evaporation duct near the shore and based on an iron tower platform was constructed using hydrological and meteorological sensors at different heights. All sensors were selected based on their high level of precision and stability at each level in the system to guarantee the accuracy and stability of the data measured. The evaporation duct can be measured by log-linear fit as well as prediction models so that the measured results can be compared to each other. A data acquisition and processing experiment utilizing this meteorological gradient instrument was conducted over the SCS, and the adaptability of the model was analyzed in this paper.

## Theory and methods

### Atmosphere refractivity and hydrometeorology

Due to the minute differences between the value of the refractive index in the troposphere (1.00003) and in free space (n = 1.0), it is more convenient to refer to variations in the refractive index in terms of a new parameter called refractivity N, which is defined as follows:

$$N=(n-1)\times 10^{-6}$$ (1)

To calculate the refractive index of the atmosphere with a single formula, the International Telecommunication Union Radio Communication Assembly (ITU-R) recommends that the atmospheric radio refractive index be computed by means of the formula provided in Eq (1).

Therefore, N is the radio refractivity expressed by

$$N=(n-1)\times 10^{6}=\frac{77.6}{T}(p+\frac{4810e}{T})$$ (2)

where T (K) is the atmospheric temperature, p (hPa) is the total atmospheric pressure, and e (hPa) is the water vapor pressure. The Debye theory has been used to describe N in terms of atmospheric pressure, water vapor pressure, and temperature [16–18]. This expression can be applied for all radio frequencies up to 100 GHz with an error of less than 0.5%. The constants are empirically derived from dielectric constant measurements and are valid for radio frequencies between 1 and 100 GHz. The modified refractivity M, which takes the earth’s curvature into account, is related to radio refractivity N and is expressed by the following:

M=N+0.157z (3)

In the above equation, z is the altitude in meters, and *M* is the modified refractivity. This expression can be applied for all radio frequencies with an error of less than 0.5%. Because the measured temperatures are very close to the true values, those calculated with modified refractivity *M* can be regarded as the true values in engineering applications.

### Paulus-Jeske model (PJ model)

**Fig 1:**
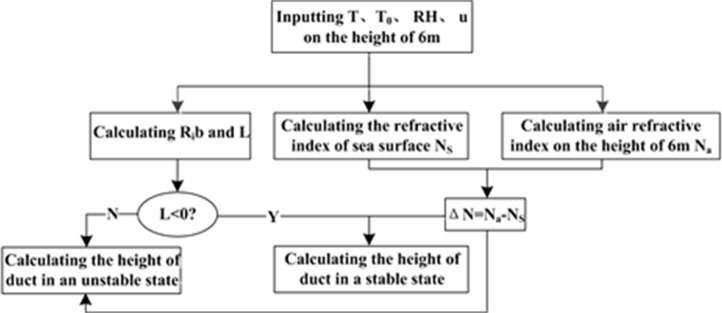
Algorithm flowchart of the PJ model.

The PJ model was widely used evaporation duct model and it has been incorporated into the IREPS microwave propagation prediction model by the United States Navy since 1978 [19]. The PJ model uses air temperature, relative humidity, wind speed at 6 m, and SST as inputs, but it assumes a constant surface atmospheric pressure of 1000 hPa. The algorithm flowchart of PJ model is shown in Fig 1.

Paulus derived the critical potential refractivity gradient for ducting as follows:

$$\frac{\partial N_{p}}{\partial z}=\frac{\partial N}{\partial z}-\frac{\partial N}{\partial p}\frac{\partial p}{\partial z}$$ (4)

where *N*_*P*_ is the potential refractivity, and *N* is the non-potential refractivity. Their research determined that this term was equal to -0.157 for ducting. Because the term varied by only 0.02 hPa^-1^ over a temperature range of 0~30°C, Paulus selected a constant value of 0.27 hPa^-1^. Combined with the hydrostatic relationship and the ideal gas law, these values are used in Eq (9). The potential refractivity gradient for ducting is -0.125. The bulk Richardson number was used to categorize the atmospheric stability and to calculate the Obukhov length in the PJ model. In stable or neutral conditions, if the calculated duct height is negative or is greater than the estimated Obukhov length L, then the PJ model considers the estimated Obukhov length to be inaccurate. Instead of using this estimated Obukhov length in the duct height calculation, the PJ model substitutes the duct height variable for the estimated Obukhov length in the PJ model’s duct height equation and solves for the duct height again. Paulus defended this procedure by stating that the ratio of the duct height to the estimated Obukhov length should never exceed unity. In most cases, the PJ model restricts duct heights from exceeding 40 m [20–22].

### NPS model

**Fig 2:**
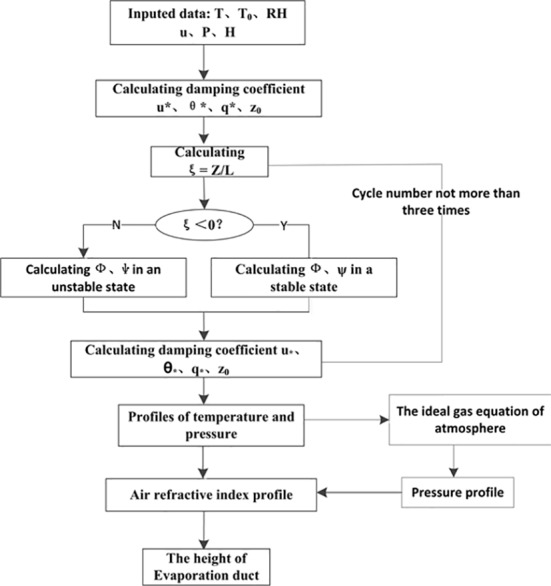
Algorithm flowchart of the NPS model.

Models such as NPS, BYC, MGB, and NWA have been studied by researchers who carried out careful theoretical analyses and used buoy data to validate and estimate these models [23–24]. Their results showed that the NPS and BYC model were better than MGB and NWA for estimating the height and the refractivity profile of evaporation ducts. The basic principles of the two models are the same except for the choice of universal function and the determination of duct height. Compared to the BYC, the NPS model provides better stability and requires only a set of sensors at a given height. The evaporation detection system of the NPS model is simpler than log-linear fit. Thus, this paper uses the NPS model to estimate the evaporation duct. The NPS model was released by the American Naval Postgraduate School in 2000. The algorithm flow chart of the NPS model is shown in Fig 2.

NPS calculates the evaporation duct based on the Monin-Obukhov Similarity Theory, which is different from the PJ model and the modified refractivity profile. The NPS model initially calculates the temperature, humidity and air pressure profiles. The expressions can be denoted as follows:

$$T(z)=T_{0}+\frac{\theta_{*}}{\kappa }[\ln(\frac{z}{z_{0t}})-\psi_{h}(\frac{z}{L})]-\Gamma_{d}z$$ (5)

$$q(z)=q_{0}+\frac{q_{*}}{\kappa }[\ln(\frac{z}{z_{0t}})-\psi_{h}(\frac{z}{L})]$$ (6)

$$p(z_{2})=p(z_{1})\exp[\frac{g(z_{1}-z_{2})}{RT_{V}}]$$ (7)

where T(z) and q(z) correspond to the air temperature and humidity at a given height; *T*_*0*_ and *q*_*0*_ correspond to the SST and humidity, respectively; θ_*_ and q_*_ are the Monin-Obukhov scaling parameters; *κ* is equal to 0.4; Γ_*d*_ is equal to 0.00976 K/m; *ψ*_*h*_ is the universal function; *z*_0*t*_ is temperature roughness height; and L is a similar length. The NPS model uses the TOGA COARE bulk flux algorithm to calculate the values of θ_*_ and q_*_. This algorithm is described in detail by Fairall et al. and is available at http://www.coaps.fsu.edu:80/coare/fluxpalgor/. R is the gas constant of dry air, and g is the gravity acceleration. *T*_*v*_ is the mean value of virtual temperature at a height of *z*_1_ and *z*_2_. The temperature, humidity and air pressure profiles can be obtained from these equations, which can subsequently determine the profile of modified refractivity. The minimum value of refractivity corresponds to the height of the evaporation duct.

## Experiment

**Fig 3:**
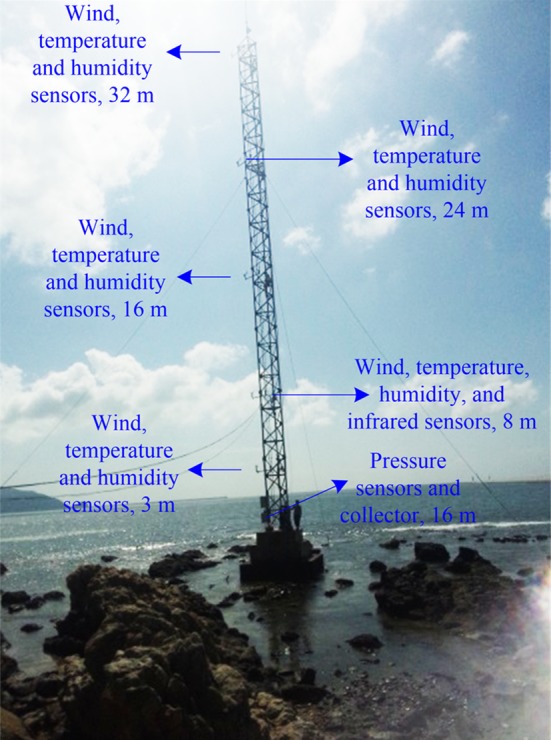
Detection system of evaporation duct based on iron tower platform.

The detection system of the evaporation duct based on the iron tower platform (N 18° 13′ E 109° 30′) is shown in Fig 3. It consists of an iron tower platform placed far from the effects of land-based collector, power, and sensor systems. The experiment was carried out from 4:57 pm, October 31, 2014 to 9:45 am, November 5, 2014. The collector received a set of sensor data every three seconds and outputted the mean value every three minutes. The experiment collected a total of 2257 data sets. Each set of data contained five groups of data: wind, air temperature, relative humidity, air pressure, and SST. The meteorological sensors were divided into five levels corresponding to 3 m, 8 m, 16 m, 24 m and 32 m above the sea surface, as shown in Fig 3. Wind and humidity sensors were installed at each level, and an infrared sensor was fixed at the second level (8 m) for measuring the SST. No systematic deviations existed between the different levels of sensors in the evaporation detection system. The sensor parameters are shown in Table 1.

**Table 1:** Meteorological sensor parameters.

Meteorological sensors	Location	Measuring range	Measuring accuracy
Wind speed (m/s)	each level	0.5~90	±0.2
Relative humidity (%)	each level	0~100	±1(0~90), ±2(90~100)
Air temperature (°C)	each level	-30~50	±0.2
Air pressure (hPa)	first level	600~1100	±1.5
SST (°C)	second level	-30~50	±0.5

The height of the evaporation duct is a key factor in determining the practical height of a radar installation. Thus, the modified refractivity M for each level was obtained from the corresponding air pressure, temperature, and water vapor pressure according to Eqs (2) and (3). Six pairs of (z, M) were obtained at heights of 0, 3, 8, 16, 24 and 32 m, and nonlinear least-squares fitting was used to calculate the M value for each 0.1 m for all cases based on a log-linear function given by the following:

$$M=f_{0}z-f_{1}\ln(z+0.001)+f_{2}$$ (8)

where the coefficients *f*_0_, *f*_1_ and *f*_2_ were calculated using a least-squares best fit. The constant 0.001 was applied to prevent the curve from failing at zero altitude.

## Results and discussion

**Fig 4:**
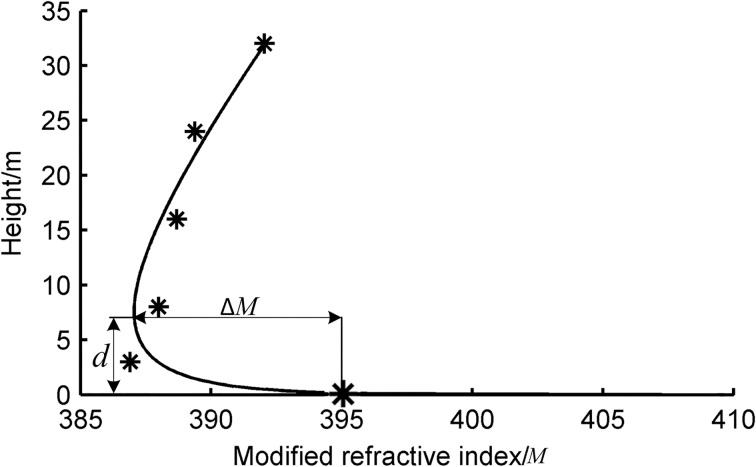
Log-linear function for M profile at a given moment.

To evaluate the height of the evaporation duct, the profile of the atmospheric modified refractivity (*M* profile) must be determined initially; the log-linear function was used to obtain the *M* profile as shown in Fig 4. It can be observed that the cure fit profile shows good agreement. The height and intensity of evaporation duct are two important parameters that correspond to *d* and Δ*M* in Fig 4. The value of *d* is obtained when the modified refractivity as a function of the height achieves a minimum value. Δ*M* is the difference of the refractivity at the height of 0 m and *d*. When *d* and Δ*M* are evaluated, the average value of a certain time period is typically authentic and believable. However, any value may appear to be nonsensical at a given moment. Therefore, statistics are used in data acquisition and processing. Several quality control methods were applied to eliminate unreasonable data. All raw data were checked using corresponding visible Figs and any inaccurate data such as singulars points outside of the measuring range or not following the gradient information were eliminated.

### Characteristics of evaporation duct observed

**Fig 5:**
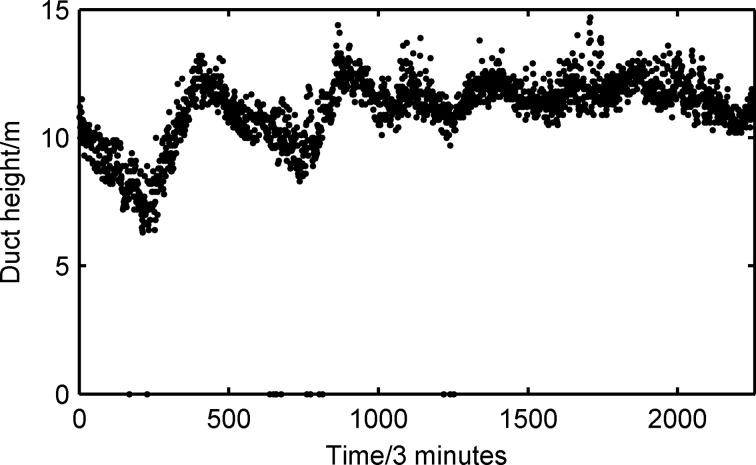
Measured values of duct height.

Fig 5 shows the measured value of the duct height calculated by log-linear fit over the five days. The height of the evaporation duct varied from 0 m to 15 m within those days, though most duct heights were concentrated between 5 and 15 m. Based on these statistical results, the occurrence probability of an evaporation duct is 89.3% in the SCS. The mean duct height for evaporation is 10.62 m with a 0.89 m Standard Deviation (STD). This value is lower than the global mean height of 13 m [18,24], but the measured result is in accordance with statistical results obtained by the National Centers for Environmental Prediction in the SCS. Water vapor at the ocean surface is saturated but becomes unsaturated with increased altitude. As a result, the water vapor levels rapidly decrease from the ocean surface upwards, thus causing a decrease in the modified refractive index with an increase in height. Eventually, the minimum value of the modified refractive index is obtained at a certain height designated the duct height. In short, the appearance of an evaporation duct owe to a body of seawater evaporation resulting in decreased humidity over several meters.

**Fig 6:**
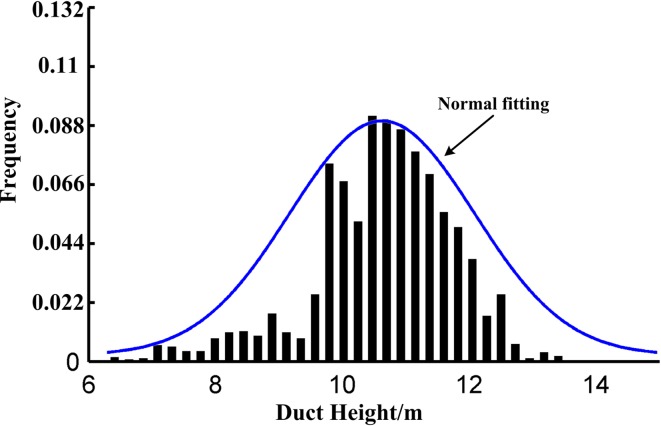
Frequency histogram of duct height.

During the five-day experiment, the weather was sunny for the first two days and wet for the other days. It was assumed that one day was an observation cycle. The duct height shown in Fig 5 dramatically decreased in the front half of the cycle. And then it significantly increased in the following half cycle. As the solar irradiation weakened, the air temperature gradually decreased and the relative humidity increased in the front cycle, thus leading to a decrease in the rate of heat exchange and a rapid decrease in the duct height. Over the other three days, the duct height exhibited slight oscillations compared with the first two days, which can be explained by the weak rate of heat exchange and smaller oscillations in relative humidity compared to the first two days. The frequency histogram of the duct height is shown in Fig 6. The horizontal axis is the evaporation duct height, and the vertical axis is the frequency of duct occurrence. The blue solid line is the normal fitting curve. It can be observed that the curve concurs with the measured results of the duct height. The proportional duct height concentrated between 10 m and 12 m and reached 75.2%. Therefore, it is appropriate to describe the height of the atmosphere duct with probability statistics.

**Fig 7:**
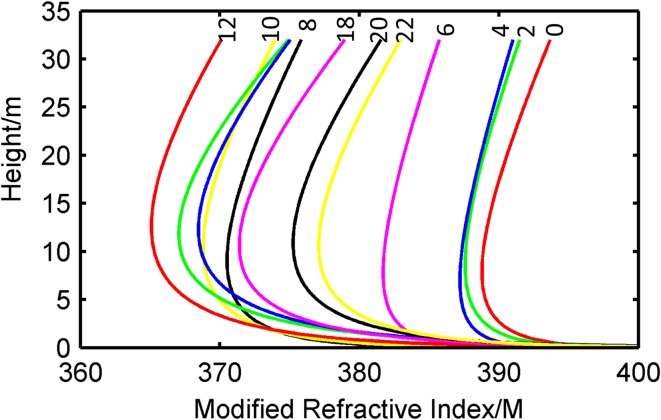
Modified refractive index of one day (Nov. 1).

**Fig 8:**
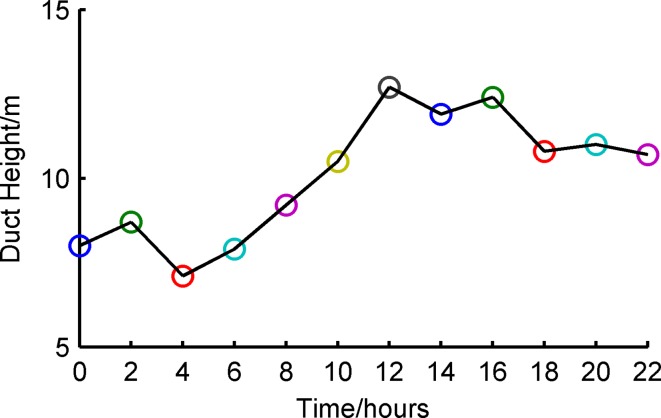
Duct height of one day (Nov. 1).

The atmosphere refractive index can determine radio wave bending paths and affect the propagation region. The *M* profile is shown in Fig 7; the horizontal axis is the modified refractive index, and the vertical axis is the height over sea level. The labeled color curve extends from 0:00 to 22:00 on November 1, 2014. Fig 8 shows the curvature of the duct height at different moments. It can be observed that the duct height and intensity increased during the first cycle and decreased in the subsequent cycle. The *M* profile was unchanged from 0:00 to 4:00 and varied acutely from 6:00 to 12:00, which could be interpreted as the rate of heat exchange between the atmosphere and seawater. The atmosphere temperature rises rapidly when the sun comes out, thus leading to an increasing rate of heat exchange between the atmosphere and seawater.

### Comparison and analysis

**Fig 9:**
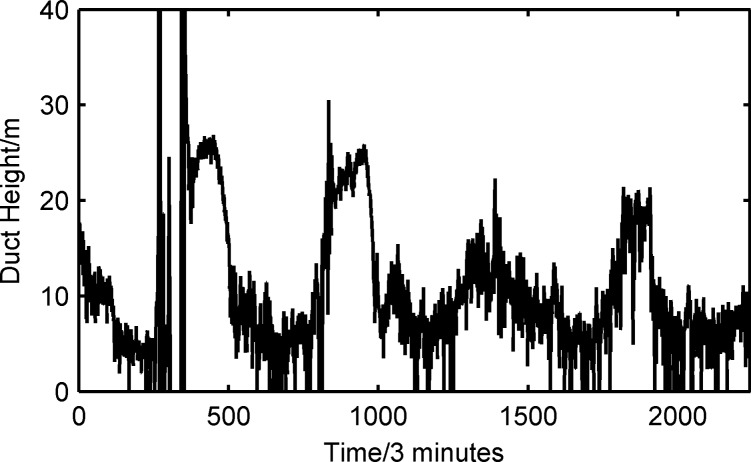
Predicted value of the duct height using the PJ model.

The predicted value of the duct height using the PJ model is shown in Fig 9. The duct height varied from 0 m to 40 m. The mean value of the duct height was 11.65 m higher than the true value. The mean deviation (MD) is the difference between the measured results and the true value. The MD was 1.03 m and the standard deviation (STD) of MD was 3.4 m compared with the true value shown in Table 2. The resulting STD of the duct height increased to 4.73 m, which was significantly greater than 0.89 m. At a certain moment, the predicted results for the PJ model showed significant differences, and the duct height achieved 40 m compared with the true value. This indicates that the predicted value of the PJ model will induce significant errors for the district of the SCS. The results also showed that the PJ model relied on a body of experiments and required copious experimental data to revise the experiential formula and ensure model accuracy. Because environmental conditions (such as sea surface waves, latitude, longitude and climate) are not identical, the adaptability of the model is not uniform. Although the PJ model addresses measurement inaccuracy, constructing a PJ model system that only requires a set of data sensors at a 6-m height is relatively simple and applied widely in practice.

**Table 2:** Duct height parameters.

Methods	Mean value	STD	MD (m)	STD of MD (m)
Log-linear fit	10.62	0.89	0	0
PJ model	11.65	4.73	1.03	3.4
NPS model	8.92	1.7	-1.4	0.8

**Fig 10:**
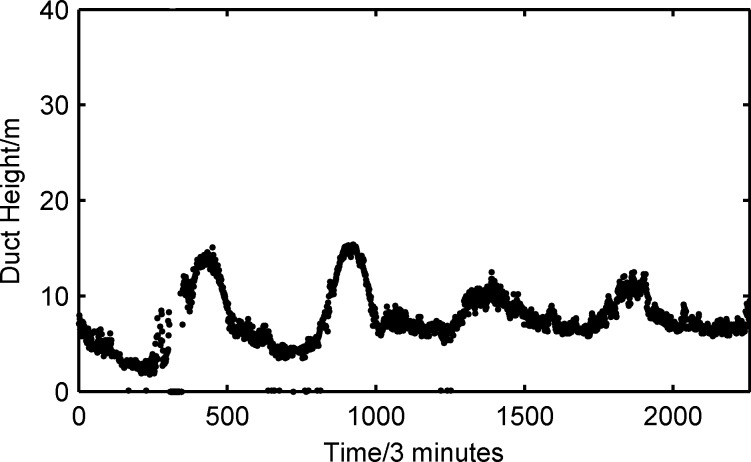
Predicted value of the duct height using the NPS model.

As for the NPS model shown in Fig 10, the evaporation height was less than 20 m. The mean value was 8.92 m, and the STD was 1.7 m. The MD was -1.4 m, and the STD of MD was 0.8 m compared with the true value shown in Table 2. The predicted value using the NPS model was less than the true value. It demonstrated better stability than the PJ model. It calculated the pressure, temperature and humidity profiles that accurately described the air refractivity and the fluctuation was slight. For the three calculated results, the curves show a good trend, but the degree of fluctuation was different. The duct height presented a trend of periodic variation similar to diurnal change as demonstrated by air pressure, temperature, and water vapor pressure and was closely related with daily change in climate. During the first two days, the weather was sunny, and the duct height as a function of time exhibited marked daily change. During the last three days, the weather was dull and raining, and the duct height amplitude was gentler and displayed stable tendencies. This indicates that weather conditions, such as sunny or wet weather, have an effect on duct height.

The predicted models were based on the Monin-Obukhov similarity theory. The similarity theory of universal function or its integral form is usually based on the test conditions of the land, which is directly applied to the detection of evaporation at sea. Perhaps the universal function is not applicable in this case. Sea surface roughness is an important physical quantity in the construction of an evaporation duct model, but there is no complete unified calculation formula for calculating sea surface roughness. The applicability of the model in this paper provides a reference for the selection of the model. Sometimes, when the errors of predicted value are calculated through one model, we can combine with two models to improve accuracy. Because of the limitation of the experimental conditions and the experimental data, this research is not comprehensive, and the applicability of the evaporation duct model not only requires more experimental data but also needs to be analyzed in different sea areas and weather conditions.

## Conclusion

This paper used PJ and NPS models based on meteorological gradient instruments to obtain evaporation duct heights, which are significant not only for research on atmospheric phenomena and climate regulation but also for providing information on atmosphere ducts in the SCS. The results showed that the NPS model was more stable than the PJ model. The NPS model may be better suited for estimating evaporation ducts in practice due to its simple structure. The experimental data were obtained in winter, and the climatic conditions do not exhibit distinct changes in the same season over tropical oceans. Thus, these experimental data and results can be useful in estimating evaporation ducts over the SCS.
